# The Establishment of a Registry of Respiratory Patients Attending Tikur Anbessa Specialized Hospital, Addis Ababa, Ethiopia: A Feasibility Study

**DOI:** 10.4314/ejhs.v35i1.4S

**Published:** 2025-12

**Authors:** Amsalu Bekele, Eyob Kebede Etissa, Dominick Shaw, Andrew W Fogarty

**Affiliations:** 1 Division of Pulmonary and Critical Care Medicine, Department of Internal Medicine, College of Health Sciences, Addis Ababa University; 2 Department of Respiratory Sciences, University of Leicester, Institute for Lung Health, NIHR Respiratory Biomedical Research Centre, Leicester, UK; 3 Department of Lifespan and Population Sciences, NIHR Biomedical Medical Research Centre, School of Medicine, University of Nottingham, UK

**Keywords:** Asthma, COPD, Characterization, Lung function

## Abstract

**Background:**

Respiratory diseases cause substantial morbidity and mortality in low- and middle-income countries (LMICs). Registries in high-income countries have significantly advanced the understanding of lung diseases. This feasibility study aimed to establish a registry of patients with suspected respiratory disease at a major teaching hospital in Addis Ababa, Ethiopia.

**Methods:**

A prospective hospital-based registry was established at Tikur Anbessa Specialized Hospital (TASH) for patients aged ≥18 years with respiratory disease. The registry collected demographic, clinical, and spirometric data.

**Results:**

The registry captured data from 285 participants. Of these, 132 (46%) were diagnosed with asthma and 41 (14%) with chronic obstructive pulmonary disease (COPD). Among patients with asthma, the median pre-bronchodilator forced expiratory volume in one second (FEV_1_) was 0.94 L, with a percent predicted of 39% (IQR: 29–55). Post-bronchodilator FEV_1_ increased to a median of 1.20 L, percent predicted 54% (IQR: 38–70), with a median percentage change in FEV_1_ of 27%. Asthma prevented 54 participants (41%) from performing basic life activities. According to the Global Initiative for Asthma (GINA) symptom assessment tool, only 6 patients (4%) had controlled asthma.

Among patients with COPD, the median pre-bronchodilator FEV_1_ was 0.97 L, percent predicted 43% (IQR: 34–61). Post-bronchodilator FEV_1_ was 1.02 L, percent predicted 46% (IQR: 37–64), with a median percentage change in FEV_1_ of 6% (IQR: 2–8).

**Conclusion:**

This study demonstrates that establishing a respiratory patient registry in Ethiopia is feasible. Such registries represent an important first step toward defining disease burden, identifying risk factors, improving treatment, and ultimately enabling tailored clinical trials to advance respiratory care in the country.

## Introduction

Respiratory diseases are among the most common causes of morbidity and mortality in low- and middle-income countries (LMICs) (1), despite being largely preventable through public health interventions or treatable with lifestyle modifications and medication. Two major respiratory diseases are asthma and chronic obstructive pulmonary disease (COPD). Asthma is characterized by reversible airflow obstruction, whereas COPD is defined by irreversible airflow limitation and damage to the lung parenchyma. Many asthma-related deaths occur in LMICs due to widespread underdiagnosis and undertreatment (2). COPD is the third leading cause of death globally, accounting for 3.23 million deaths in 2019, with more than 80% occurring in LMICs (3).

Asthma and COPD continue to impose a disproportionately high burden of morbidity and mortality in LMICs. These diseases are strongly associated with poverty, infectious diseases, and other non-communicable diseases, resulting in comorbidities that significantly affect the lives and livelihoods of affected individuals. Furthermore, the availability and cost of diagnostic tools and medications recommended for asthma and COPD management remain major challenges in sub-Saharan Africa (SSA) (4,5).

In Ethiopia, indoor air pollution related to rural living conditions and the use of biomass fuels for cooking and heating is a leading cause of COPD and other airway diseases. Tobacco smoking has also emerged as a public health concern in urban areas. Chronic respiratory diseases accounted for 1.6% of all disability-adjusted life years (DALYs) lost in Ethiopia in 2017. Nonetheless, several local studies have reported asthma to be a relatively common condition, affecting approximately 1.5–3% of the population. The prevalence of COPD in Ethiopia remains unclear.

In high-income countries, the establishment of respiratory disease registries has transformed the understanding of these conditions. Such registries have strengthened the evidence base regarding disease pathophysiology and enabled the design and implementation of effective public health interventions to reduce disease burden. Additionally, registries have facilitated the evaluation of new treatments in disease-specific populations and the identification of clinically relevant phenotypes. For example, in cystic fibrosis, life expectancy has increased dramatically from approximately 15 years in 1970 to more than 50 years for a child born today (6). However, these registries—and their associated benefits—are largely absent in Ethiopia.

Therefore, this study aimed to assess the feasibility of establishing a respiratory patient registry at a busy respiratory clinic in Addis Ababa, Ethiopia.

## Methods and Materials

**Study design**: A prospective, hospital-based registry was established to systematically characterize respiratory patients at Tikur Anbessa Specialized Hospital (TASH). Comprehensive clinical data were collected from consecutive patients over a 12-month period (September 1, 2020, to August 30, 2021). The registry captured demographic characteristics, diagnostic parameters including pre- and post-bronchodilator spirometry, treatment patterns, and disease control metrics using standardized case report forms.

**Study setting and period**: The study included respiratory patients attending the chest unit at TASH between September 1, 2020, and August 30, 2021. TASH, located in Addis Ababa, Ethiopia, is the largest tertiary hospital in the country and serves approximately 500,000 patients annually. The chest unit receives more than 500 visits per month from patients with various respiratory conditions, including asthma, COPD, previously treated pulmonary tuberculosis, and other chronic lung diseases. The unit is equipped with spirometry, bronchoscopy services, pleural biopsy, and point-of-care ultrasound.

**Source population:** All respiratory patients attending the chest clinic at TASH during the study period constituted the source population.

**Study population**: All respiratory patients attending the chest clinic during the study period who met the inclusion criteria were enrolled.

**Sample size determination**: All patients diagnosed with asthma or COPD during the one-year study period were included.

**Study variables**: Patients attending the TASH chest clinic were invited to participate. Those who consented provided socio-demographic data, risk factors, disease history, duration of illness, medication use, history of exacerbation, spirometry results, and asthma control status. Data was updated at each clinic visit, and information from the most recent visit was used for analysis.

**Inclusion and exclusion criteria**: Adult respiratory patients aged ≥18 years attending the TASH chest clinic were included. Patients younger than 18 years, those unable to perform spirometry, or those unwilling to participate were excluded.

**Data collection procedure and quality assurance:** Data on socio-demographic characteristics, asthma and COPD history, exacerbations, medication use, and disease control status were collected by trained nurses. Spirometry was performed by a nurse trained in pulmonary function testing.

Asthma control status was assessed using the Global Initiative for Asthma (GINA) symptom control tool, which evaluates daytime symptoms, night waking, reliever use, and activity limitation over the preceding four weeks. Asthma was classified as well controlled if none of these features were present, partly controlled if one or two were present, and uncontrolled if three or more were present (7).

### Pulmonary function measurement

Spirometry was performed using a Diagnostic EasyOne Plus model 2001 SN spirometer by a trained nurse. Acceptability and reproducibility were assessed according to European Respiratory Society and American Thoracic Society standards (8). Forced expiratory volume in one second (FEV_1_) and forced vital capacity (FVC) were measured during clinical stability after patients withheld beta-agonists for at least four hours. Bronchodilator reversibility testing was performed by administering four puffs (400 µg) of salbutamol (100 µg per puff) via a Volumatic spacer, followed by repeat spirometry after 15 minutes.

Asthma was diagnosed using GINA criteria, defined as a pre-bronchodilator FEV_1_/FVC ratio <0.80 with an increase in FEV_1_ of ≥12% and ≥200 mL following bronchodilator administration (7). COPD was diagnosed according to Global Initiative for Chronic Obstructive Lung Disease (GOLD) criteria, defined as a post-bronchodilator FEV_1_/FVC ratio <0.70 with no significant reversibility, indicating persistent airflow limitation (9).

**Data management and analysis**: Data were checked and cleaned before entry into Microsoft Excel and analyzed using SPSS version 25. Descriptive analyses were conducted to characterize the study population. Categorical variables were summarized using frequencies and percentages, while continuous variables were reported as medians and interquartile ranges (IQRs) due to non-normal distributions. Pre- and post-bronchodilator FEV_1_ values were compared using the Wilcoxon signed-rank test. A p-value <0.05 was considered statistically significant.

**Ethical considerations**: Ethical approval was obtained from the Department of Internal Medicine and the Institutional Review Board of the College of Health Sciences, Addis Ababa University. Data was collected anonymously, and all participants provided written informed consent.

## Results

A total of 320 participants were enrolled in the registry, of whom 285 (89%) underwent spirometry. Among these, 132 (46%) were diagnosed with asthma, 41 (14%) with COPD, 92 (32%) had restrictive lung physiology, and 20 (7%) had normal spirometry findings.

Overall, 111 participants (39%) were aged 60 years or older, and 168 (59%) were female. The median age was 55 years (IQR: 40-63). Most participants (256; 90%) resided in Addis Ababa, and 168 (59%) were unemployed. The median body mass index (BMI) was 24.8 kg/m^2^ (IQR: 21.4–27.8); 89 participants (31%) were overweight and 48 (17%) were obese.

Only two participants were current smokers, while 17 (6%) were former smokers. The remaining 266 participants (93%) never smoked. Seventy-nine participants (27.7%) reported exposure to pets, most commonly dogs and cats (Table 1).

**Table 1:** Socio-demographic and risk factors

Characteristics		All n=285(%)	Asthma n=132(%)	COPD n=41(%)
Age: Median (IQR)		55 (40, 63)	57 (45, 64)	60 (47, 65)
Age	15-29	22 (7.7)	4 (3.0)	3 (7.3)
	30-39	44 (15.4)	17 (12.9)	4 (9.8)
	40-49	33 (11.6)	17 (12.9)	3 (7.3)
	50-59	75 (26.3)	39 (29.5)	9 (22.0)
	≥60	111 (38.9)	55 (41.7)	22 (53.7)
Sex	Male	117 (41.1)	63 (47.7)	18 (43.9)
	Female	168 (58.9)	69 (52.3)	23 (56.1)
Address	Addis Ababa	256 (89.8)	121 (91.7)	35 (85.4)
	Out of Addis Ababa	29 (10.2)	11 (8.3)	6 (14.6)
Occupation	Unemployed	168 (58.9)	76 (57.6)	30 (73.2)
	Government	47 (16.5)	20 (15.2)	4 (9.8)
	Labor work	37 (13.0)	17 (12.9)	2 (4.9)
	Teacher	9 (3.2)	7 (5.3)	1 (2.4)
	Merchant	9 (3.2)	6 (4.5)	0 (0.0)
	Construction work	4 (1.3)	2 (1.5)	1 (2.4)
	Farmer	3 (1.1)	1 (0.8)	1 (2.4)
	Other	8 (2.8)	3 (2.3)	2 (4.9)
BMI	Underweight	28 (9.8)	12 (9.1)	7 (17.1)
	(<18.5 Kg.m2)			
	Normal (18.5-24.9 Kg.m2)	120 (42.1)	58 (43.9)	20 (48.8)
	Overweight	89 (31.2)	43 (32.6)	9 (22.0)
	(25-29.9 Kg.m2)			
	Obesity (≥30 Kg.m2)	48 (16.8)	19 (14.4)	5 (12.2)
Smoking	Current smoker	2 (0.7)	2 (1.5)	0 (0.0)
	Ex-smoker	17 (6.0)	4 (3.0)	4 (9.8)
	Never smoker	266 (93.3)	125 (94.6)	37 (90.2)
Any pets	Yes	79 (27.7)	36 (27.2)	12 (29.3)
	No	206 (72.3)	95 (71.9)	29 (70.0)
Types of pets	Dog	38 (13.3)	19 (14.5)	3 (7.3)
	Cat	39 (13.7)	17 (13.1)	8 (19.5)
	Chicken	5 (1.8)	4 (3.1)	0 (0.0)
	Sheep	6 (2.1)	4 (3.1)	1 (2.4)

Overall, 90% of participants were treated with salbutamol. Inhaled corticosteroids (beclomethasone) were used by 118 patients (41%), while 89 (31%) received combination inhalers (Symbicort). Oral corticosteroids were prescribed to 59 participants (21%), with 3 patients receiving long-term systemic steroids and 1 patient receiving theophylline.

During the preceding 12 months, 187 participants (66%) required two to three courses of oral corticosteroids, and 104 (36%) sought emergency care. Wheezing was reported by 195 participants (68%) in the past 12 months and by 168 (59%) in the preceding month. One death occurred during the one-year study period (Table 2).

**Table 2:** History, exacerbation and control status of participants

Characteristics	All n=285(%)	Asthma n=132(%)	COPD n=41(%)
Age of onset: Median (IQR)	16 (6, 30)	20 (10, 30)	20 (10, 35)
Family history of asthma			
Yes	108 (37.9)	46 (34.8)	16 (39.0)
No	177 (62.1)	86 (65.2)	25 (61.0)
History of Eczema/Allergic Rhinitis			
Yes	179 (62.8)	77 (58.3)	28 (68.3)
No	106 (37.2)	55 (41.7)	13 (31.7)
Current asthma medication			
Salbutamol	257 (90.2)	122 (92.4)	37 (90.2)
Inhaled steroids, (Beclomethasone)	118(41.4)	51 (38.6)	18 (43.9)
Combination inhaler, (Symbicort)	89 (31.2)	51 (38.6)	15 (36.6)
Oral steroids	59 (20.7)	29 (22.0)	6 (14.6)
Long term steroids	3 (1.1)	1 (0.8)	1 (0.8)
Theophylline	1 (0.4)	1 (0.8)	0 (0.0)
Courses of oral steroids in the past 12 months			
≤1	81 (28.4)	34 (25.8)	8 (19.5)
2-3	187 (65.6)	90 (68.2)	31 (75.6)
≥4	17 (6.0)	8 (6.0)	2 (4.9)
Emergency care in the last 12 month			
Yes	104 (36.5)	53 (40.2)	13 (31.7)
No	181 (63.5)	79 (59.8)	28 (68.3)
Wheeze in the last 12 month			
Yes	195 (68.4)	88 (66.7)	29 (70.7)
No	90 (31.6)	44 (33.3)	12 (29.3)
Wheeze in the last month			
Yes	168 (58.9)	76 (57.6)	22 (53.7)
No	117 (41.1)	56 (42.4)	19 (46.3)
Outcome			
Alive	284 (99.1)	132 (100.0)	41 (100.0)
Dead	1 (0.9)	0 (0.0)	0 (0.0)

The median FEV1 was 1.05L (IQR: 0.77 to 1.60), percent predicted was 50% (IQR: 35 to 63) while the post bronchodilator FEV1 was 1.21L (IQR: 0.83 to 1.64), percent predicted 56% (IQR: 41 to 69), and median % change in FEV1 was 17% (IQR: 7.22, 33.67). The median pre FEV1/FVC was 0.68 (IQR: 0.54 to 0.78)) and the post FEV1/FVC was 0.68 (IQR: 0.57 to 0.77) (Table 3).

**Table 3:** Lung function of study participants (**N=285**)

Characteristics	All Median (IQR)	Asthma n=132	COPD n=41
FEV1 before bronchodilator	1.05 (0.77, 1.60)	0.94 (0.61, 1.21)	0.97 (0.77, 1.28)
% Pred FEV1 before bronchodilator	50 (35,63)	39.50 (28.75, 55.25)	43 (34,61)
FEV1 after bronchodilator n=244	1.21 (0.83, 1.64)	1.20 (0.78, 1.62)	1.02 (0.70, 1.37)
% Pred FEV1 after bronchodilator n=244	55.59 (41.0, 69.0)	54 (37.75, 70)	46 (37.50, 64)
FEV1/FVC before bronchodilator	0.68 (0.54-0.78)	0.55 (0.48, 0.63)	0.65 (0.59, 0.69)
FEV1/FVC after bronchodilator n=244	0.68 (0.57, 0.77)	0.60 (0.51,0.67)	0.65 (0.58, 0.73)
% Change in FEV1 n=244	17.55 (7.22, 33.67)	26.65 (19.0, 41.72)	6.30 (-2.00, 7.70)

**Asthma patients:** Asthma was diagnosed in 132 patients (46%), with a median age of 57 years (IQR: 45–64). Thirty-six patients (27%) reported exposure to pets. The median age at asthma diagnosis was 20 years (IQR: 10–30). A family history of asthma was present in 46 patients (35%), and 77 (58%) reported comorbid eczema or allergic rhinitis.

Regarding treatment, 122 patients (92%) were using salbutamol, 51 (39%) were on inhaled corticosteroids (beclomethasone), 51 (39%) were using combination inhalers (Symbicort), and 29 (22%) were receiving oral corticosteroids. One patient was on long-term systemic corticosteroids, and one patient was receiving theophylline.

Over the preceding 12 months, 90 patients (68%) required two to three courses of oral corticosteroids, 53 (40%) sought emergency care, and 88 (67%) experienced wheezing. In the month preceding assessment, 76 patients (58%) reported wheezing. Asthma limited basic daily activities in 54 patients (41%).

Spirometric evaluation showed a median pre-bronchodilator FEV_1_ of 0.94 L (IQR: 0.61–1.21), corresponding to 39% of the predicted value (IQR: 29–55). The median post-bronchodilator FEV_1_ increased to 1.20 L (IQR: 0.78–1.62), or 54% of the predicted value (IQR: 38–70). The median pre-bronchodilator FEV1/FVC ratio was 0.55 (IQR: 0.48–0.63), increasing to 0.60 (IQR: 0.51–0.67) post-bronchodilator. The median percentage change in FEV_1_ was 27% (IQR: 19–42).

Based on the Global Initiative for Asthma (GINA) symptom assessment tool over the preceding four weeks, 69 patients (52%) had uncontrolled asthma, 57 (43%) had partly controlled asthma, and only 6 patients (4%) had controlled asthma (Table 4).

**Table 4:** Asthma specific exacerbation and control status of participants (No=132)

Characteristics	Asthma No (%)
Has asthma stops you from basic life activity	
Yes	54 (40.9)
No	78 (59.1)
In the past 4 weeks the patient had	
Controlled	6 (4.5)
Partly controlled	57 (43.2)
Uncontrolled	69 (52.3)
Day time symptoms more than twice/week	
Yes	85 (64.4)
No	47 (35.6)
Any night waking due to asthma	
Yes	85 (64.4)
No	47 (35.6)
Reliever needed more than twice/week	
Yes	114 (86.4)
No	18 (13.6)
Any activity limitation due to asthma	
Yes	50 (37.9)
No	82 (62.1)

**COPD patients:** Forty-one patients (14%) were diagnosed with COPD, with a median age of 60 years (IQR: 47–65). Four patients (10%) were former smokers. Salbutamol was the most prescribed medication, used by 37 patients (90%), followed by inhaled corticosteroids (beclomethasone) in 18 (44%), combination inhalers (Symbicort) in 15 (37%), oral corticosteroids in 6 (15%), and long-term systemic corticosteroids in 1 patient (1%).

During the preceding 12 months, approximately three-quarters of patients with COPD received two to three courses of oral corticosteroids, 13 patients (32%) required emergency care, and 29 (71%) reported wheezing. In the month prior to the assessment, 22 patients (54%) experienced wheezing.

Spirometry revealed a median pre-bronchodilator FEV_1_ of 0.97 L (IQR: 0.77–1.28), corresponding to 43% of predicted (IQR: 34–61). Post-bronchodilator FEV_1_ was 1.02 L (IQR: 0.70–1.37), or 46% of predicted (IQR: 37–64). The median pre-bronchodilator FEV_1_/FVC ratio was 0.65 (IQR: 0.59–0.69), which remained unchanged post-bronchodilator at 0.65 (IQR: 0.58–0.73). The median percentage change in FEV_1_ was 6% (IQR: 2–8).

There was a statistically significant median difference between pre- and post-bronchodilator FEV_1_ among patients with asthma (p < 0.001), but not among patients with COPD (Table 5).

**Table 5:** Pre- and post-bronchodilator FEV1 Wilcoxon signed rank test comparison for Asthma and COPD patients

Variable	FEV1 Pre-bronchodilator	FEV1 post-bronchodilator	Change	p-value
Asthma n=132, Median (IQR)	0.94 (0.61, 1.21)	1.20 (0.78, 1.62)	0.27 (0.17,0.36)	0.000*
COPD n=41, Median (IQR)	0.97 (0.77, 1.28)	1.02 (0.70, 1.37)	0.05 (-0.01, 0.09)	0.122

## Discussion

These data demonstrate the feasibility of establishing one of the first respiratory disease registries in Ethiopia and serve as proof of concept that high-quality clinical and lung function data can be systematically collected when appropriate clinical expertise and spirometry capacity are available. The key findings indicate that most patients with asthma or COPD experience poorly controlled disease, frequent exacerbations, repeated emergency visits, and a markedly reduced quality of life. This burden appears to be closely linked to suboptimal treatment practices, as relatively few patients with asthma received standard inhaled corticosteroids, and none of the patients with COPD were receiving recommended maintenance therapies—an issue commonly reported in low-resource settings (10,11).

In Ethiopia, a substantial treatment gap persists, characterized by the overuse of short-acting beta-agonists (SABAs) and oral corticosteroids, alongside the underuse of inhaled corticosteroids (ICS) and combination therapies (ICS–LABA). This imbalance is likely a primary driver of high exacerbation rates and poor asthma control. Similar treatment patterns have been documented across multiple studies in the country and reflect broader systemic challenges faced by healthcare systems in resource-limited settings (3,7,12,13).

The findings reveal poor asthma control in the context of inadequate long-term management. Over a 12-month period, patients experienced frequent severe symptoms, required repeated courses of oral corticosteroids, made regular emergency visits, and reported significant limitations in daily activities. There was a clear overdependence on rapid-relief medications (SABAs) and limited use of essential preventive therapies (ICS). This suboptimal treatment approach likely contributes to the increased need for systemic corticosteroids and the nearly threefold higher rate of emergency care utilization (38%) compared with patients receiving guideline-based care (12%) (14–16).

Importantly, spirometry data extend the narrative beyond poor symptom control, revealing a high burden of severe obstructive lung disease with likely fixed airflow limitations in many patients. This finding highlights the long-term consequences of delayed or inadequate treatment, as many individuals appear to have developed irreversible lung damage that might have been prevented through early diagnosis and sustained, guideline-based therapy. These results underscore the urgent need to expand access not only to appropriate inhaler therapy but also to spirometry services to enable accurate diagnosis, phenotyping, and targeted management. Similar findings have been reported in other studies from Ethiopia and comparable low-resource settings, pointing to a broader systemic failure in chronic respiratory disease care (17-20).

Poor disease control is likely driven by multiple systemic barriers, including financial constraints, high medication costs, and gaps in guideline implementation. Notably, previous studies have shown that only about one-third of healthcare providers consistently use Global Initiative for Asthma (GINA) protocols, further compounding these challenges (7).

This study also identified a markedly higher prevalence of asthma among urban residents compared with rural populations (92% vs. 8%). These findings are consistent with epidemiological studies from Brazil (21) and Ethiopia (22) and support the well-documented urban-rural disparity in asthma burden. This pattern may be explained by chronic exposure to urban air pollution, including traffic-related emissions, industrial pollutants, and elevated ambient particulate matter concentrations in Addis Ababa (23–25).

The median age of asthma onset in this cohort was 20 years. Key risk factors included a family history of asthma in 38% of participants and a personal history of atopy, such as eczema or allergic rhinitis, in 63%. Family history is a well-established risk factor for asthma, with reported prevalence rates ranging from 2% to 26% among individuals with affected relatives (26). Our findings reinforce existing evidence that having first-degree relatives with asthma substantially increases disease risk (26).

Overall, these data present a compelling case for urgent action to improve the care of chronic respiratory diseases in Ethiopia. The focus must shift from reactive management of acute exacerbations to prevention through early diagnosis, regular follow-up, and sustained controller therapy. Investment in these strategies is not only a moral imperative to reduce preventable morbidity and mortality but also an economic necessity to build a more efficient and sustainable healthcare system. The high prevalence of severely reduced lung function observed in this study serves as a warning sign that demands immediate attention to avert escalating disability and healthcare costs.

This study has limitations. As a hospital-based study conducted at a tertiary care center, the findings may not be fully generalizable to primary care settings or the broader community. In addition, data on exposure to biomass fuels, occupational hazards, and comorbidities were limited.

In conclusion, this study demonstrates that establishing a respiratory disease registry in Ethiopia is feasible and reveals substantial gaps in the management of asthma and COPD at the tertiary care level. These findings highlight an urgent need for systemic improvements in diagnosis, treatment, and long-term management. Coordinated action involving clinicians, health system planners, and policymakers is essential to address this growing public health challenge.
